# Influence of Site, Surgery, and BMI Binary Classification on Positioning Errors in Radiotherapy for Breast Cancer Patients: A Retrospective Study

**DOI:** 10.1002/cnr2.70343

**Published:** 2025-09-13

**Authors:** Bao Wan, Fu‐Kui Huan, Yan‐Dong Ge, Rui‐Ao Zhao, Yong‐Tai Zheng, Meng Liang, Yan‐Xin Zhang, Wei Zhang, Lu Hou, Ye Zhang, Hong‐Kai Wang

**Affiliations:** ^1^ Department of Radiation Oncology National Cancer Center/National Clinical Research Center for Cancer/Cancer Hospital, Chinese Academy of Medical Sciences and Peking Union Medical College Beijing China

**Keywords:** BMI, breast cancer, positioning error, surgery type, tumor site

## Abstract

**Purpose:**

To explore whether tumor site, surgery type, and the different body mass index (BMI) of patients with breast cancer affect the positioning error.

**Methods:**

A retrospective study of 213 patients treated for breast cancer was binarily classified into groups based on the tumor site (left or right), surgery type (modified radical mastectomy, MRM and breast‐conserving surgery, BCS) and BMI criterion (24 kg/m^2^). Positioning errors were obtained by using Cone‐beam CT (CBCT) and analyzed to calculate the differences in 6 degrees of freedom. An independent sample *t*‐test for positioning error was performed with a statistically significant level of 0.05.

**Results:**

For tumor site, significant differences were observed in the *X*, *Y*, *R*_*x*_, *R*_*y*_, *R*_*z*_ directions. The *t*‐test shows a significant difference in the *X* , *Y* and *Z* directions for translation errors of different surgery types. In terms of rotational errors, the *R*_*y*_ direction shows that the MRM group has significantly lower errors than the BCS group. Considering the influence of BMI, there is a significant difference in positioning errors only in the *Z* direction.

**Conclusion:**

For breast cancer patients, the *R*_*z*_ can serve as a reference for identifying positioning errors in other directions. For patients undergoing BCS, more individualized correction strategies during positioning are necessary. For obese patients, optimization measures should be implemented to address vertical positioning errors, considering body shape and size changes during treatment.

## 1 Introduction

Breast cancer is the most high‐incidence tumor in China. With the intensification of population aging and changes in lifestyle, the incidence of breast cancer in China has been increasing in recent years, which leads to an important issue for women's health [1]. There are many factors that can cause breast cancer, and obesity is one of the main risk factors for breast cancer [2]. With the advancement of medical technology, machine‐induced errors related to gantry angles, beam sizes, and other factors have been improved to a certain extent, and are generally considered to be minor errors compared to positioning errors [3, 4]. However, the impact of body mass index (BMI) on treatment precision cannot be ignored. Some researchers have found that a higher BMI can lead to an increased risk of breast cancer incidence, recurrence, and mortality [5]. Higher BMI not only increases the risk of breast cancer but also affects the precision of positioning during radiotherapy. Liang et al. [6] reported that individuals with a larger BMI have significant skin tension after lying down, especially those with loose skin and thicker abdominal fat, which can severely affect the accuracy of positioning. In breast cancer radiotherapy, differences in the treatment site (left/right breast) significantly impact the selection and implementation of treatment strategies. For left‐sided breast cancer patients, due to the proximity of the breast tissue to the heart, radiotherapy is commonly combined with deep inspiration breath‐hold (DIBH) techniques to achieve cardiac dose constraints [7]. In contrast, right‐sided breast cancer treatment tends to involve more uncertainty, as there are fewer specific strategies available to assist in treatment planning. Furthermore, the type of surgery (modified radical mastectomy, MRM, or breast‐conserving surgery, BCS) [8] can result in distinct changes in the morphology of the breast tissue, which in turn affects the stability and reproducibility of patient positioning during treatment. Despite the potential impact of these factors on setup errors, research regarding the influence of treatment site (left/right) and surgical approach (MRM/BCS) on radiotherapy setup errors remains relatively scarce. Therefore, this study aims to analyze the impact of BMI, treatment site (left/right breast), and surgery type (MRM/BCS) on radiotherapy setup errors.

## 2 Materials and Methods

### 2.1 Clinical Data and Inclusion Criteria

Two hundred thirteen breast cancer patients who underwent radiotherapy at the Cancer Hospital of the Chinese Academy of Medical Sciences from 2016 to 2023 were selected, including 107 left breast cases and 106 right breast cases. Based on the BMI classification criteria recommended by Chinese guidelines, patients in this study were categorized into a normal weight group (BMI < 24 kg/m^2^) and an overweight group (BMI ≥ 24 kg/m^2^) [9]. All eligible patients had well upper limb abduction and external rotation function on both sides, which could meet the requirements of arm support and grip. Pretreatment CBCT scan was conducted for each patient with a frequency of 3–5 consecutive times in the first week, and once a week thereafter. All patients signed the informed consent form. The demographic information is shown in Table 1.

**TABLE 1:** General information of patients in this study.

Classifications	Type	Age	Height	Weight	Number	CBCT sets
Site	Left	49.1 (29–82)	1.61 (1.50–1.74)	62.4 (38–98)	107	815
	Right	50.2 (28–73)	1.61 (1.51–1.77)	62.0 (45–90)	106	818
Surgery	MRM	49.0 (33–73)	1.60 (1.51–1.77)	62.7 (38–98)	110	798
	BCS	50.1 (28–82)	1.62 (1.50–1.77)	61.7 (45–90)	103	835
BMI (kg/m^2^)	< 24	48.9 (28–82)	1.62 (1.52–1.77)	56.6 (38–68)	112	846
	≥ 24	50.2 (29–73)	1.61 (1.50–1.74)	68.6 (55–98)	101	787

### 2.2 CT Simulation Positioning

All patients utilized a cervicothoracic fixation board for positioning, with both arms raised and placed on armrests and wrist rests, achieving approximately 120° abduction of the upper arms. The handgrip was positioned according to the length of the patient's arms, and the headrest was adjusted based on the patient's height and neck length. A customized cervicothoracic membrane with an opening for the affected breast area was used to ensure full exposure of the breast. After the thermoplastic mask cooled and set, the midline and positioning marks were drawn on the patient's body. The midlines on both sides were aligned with the fixation board at a distance of 30 cm in the direction of the treatment couch movement, and the abdominal midline was extended to the umbilicus. Simulation scans were performed using either a Philips Brilliance Big Bore or a Siemens SOMATOM Definition AS 40 radiotherapy CT scanner under the same conditions (5 mm slice thickness and interval, covering a range from 5 cm above the thoracic inlet to 5 cm below the lung base).

### 2.3 CBCT Image Acquisition and Positioning Errors Recording

Patients received 3–5 cone‐beam computed tomography (CBCT) scans during the first week, followed by weekly CBCT scans thereafter. For patients with a single‐session displacement error exceeding the threshold, setup correction was performed with the approval of the supervising physician, followed by two additional CBCT scans. The CBCT registration region is defined with the upper boundary 2 cm above the tracheal cartilage and the lower boundary at the inferior edge of the contralateral breast. The anterior boundary extends beyond the glandular tissue or chest wall, while the posterior boundary encompasses the entire vertebral body. After automatic gray‐value registration between the CBCT and planning CT images, manual fine‐tuning was conducted. The entire registration process was completed by the same experienced radiation therapist. The six degrees of freedom (6DoF) setup errors were recorded, including three translational directions: lateral (Lat, *X*), longitudinal (Lng, *Y*), vertical (Vrt, *Z*), and three rotational directions: pitch (*R*_*x*_), roll (*R*_*y*_), and yaw (*R*_*z*_). If the setup error exceeded 1 mm, couch shifts were applied for correction.

### 2.4 Statistical Methods

SPSS 25.0 statistical software was used for data analysis. The set‐up error data were expressed as mean ± STD, and the distribution was further evaluated by the quartile of each group of data. Kolmogorov‐Smirnov normality test was performed for the six‐dimensional positioning error. When the data are normally distributed, the independent sample *t* test is used; if it does not conform to the normal distribution, Mann‐Whitney *U* test is used. The statistical significance level was set to 0.05.

## 3 Results

### 3.1 Positioning Errors of Breast Cancer Sites

**FIGURE 1:**
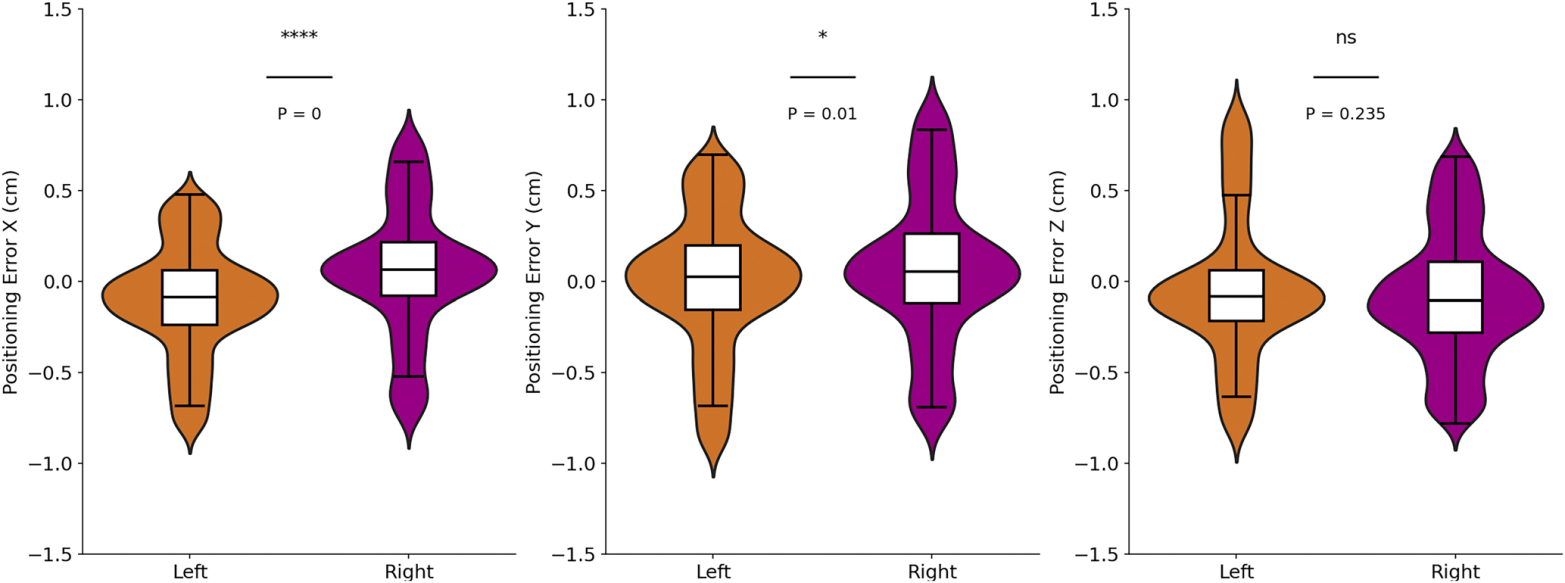
The translational error distribution of patients with different tumor sits.

**FIGURE 2:**
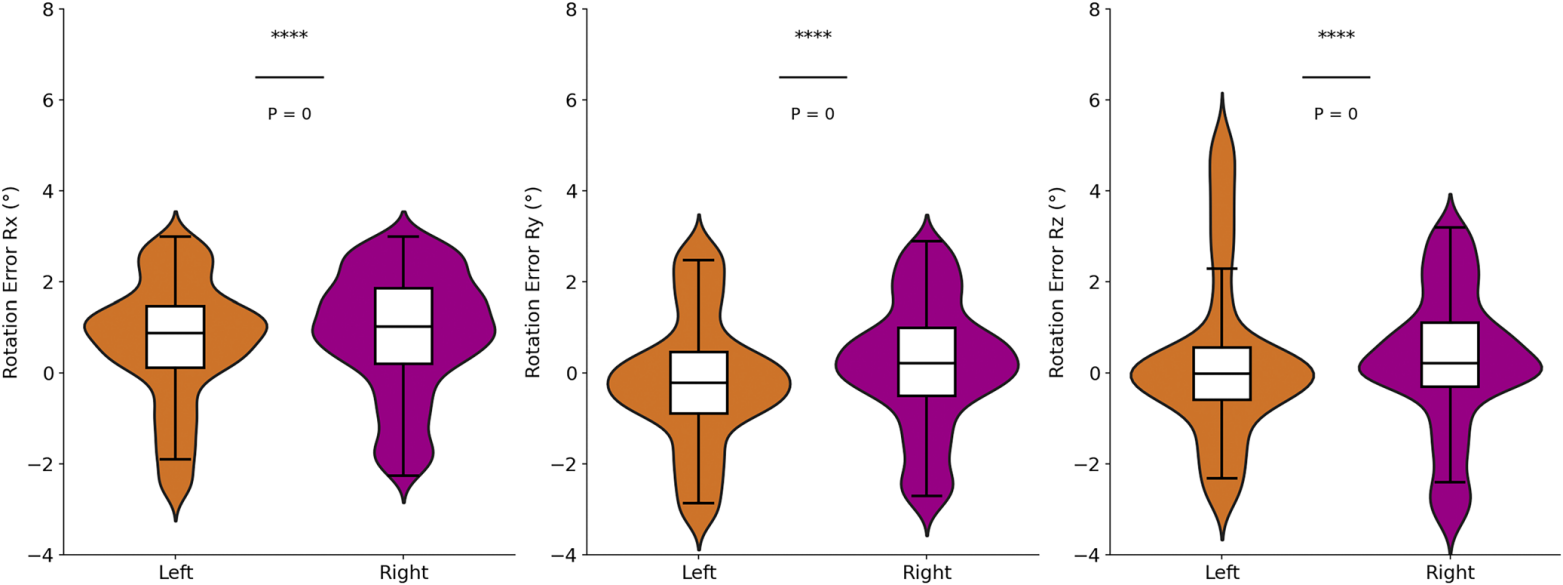
The rotational error distribution of patients with different tumor sites.

Figure 1 compares the statistical results of positioning errors between patients with left and right site breast cancer. Regarding translational errors, the range of errors for both groups is similarly distributed and concentrated within ±1 mm, demonstrating high stability in translational positioning accuracy for both patient groups, with no significant deviations observed. In the *X* and *Y* directions, the data show significant differences, with the overall distribution of the right group being slightly higher than that of the left group. For rotational errors, the right‐sided group demonstrates a more pronounced positive deviation in all three rotational directions, while the left‐sided group shows a negative deviation only in the *R*_*y*_ direction, with *R*_*z*_ remaining close to zero, as shown in Figure 2.

The *t*‐test results (Table 2) indicate significant differences in the *X*, *Y*, *R*_*x*_, *R*_*y*_, and *R*_*z*_ directions, with the most notable differences observed in the *X*, *R*_*y*_ and *R*_*z*_ directions. In contrast, the *p* value for the *Z* direction is far greater than 0.05, suggesting that the error distributions in this direction are consistent between the two groups.

**TABLE 2:** Statistical analysis results of different breast tumor sites in six DOFs.

DIR	Left		Right		Statistics	
	Mean	STD	Mean	STD	*t*	*p*
*X* (cm)	−0.079	0.240	0.048	0.227	−11.02	< 0.05
*Y* (cm)	0.018	0.276	0.053	0.273	−2.58	< 0.05
*Z* (cm)	−0.077	0.239	−0.092	0.264	1.19	0.24
*R*_*x*_ (°)	0.81	0.990	1.00	1.027	−3.84	< 0.05
*R*_*y*_ (°)	−0.15	1.112	0.21	1.063	−6.91	< 0.05
*R*_*z*_ (°)	0.03	1.072	0.32	1.019	−5.65	< 0.05

### 3.2 Positioning Errors of Surgery Types

**FIGURE 3:**
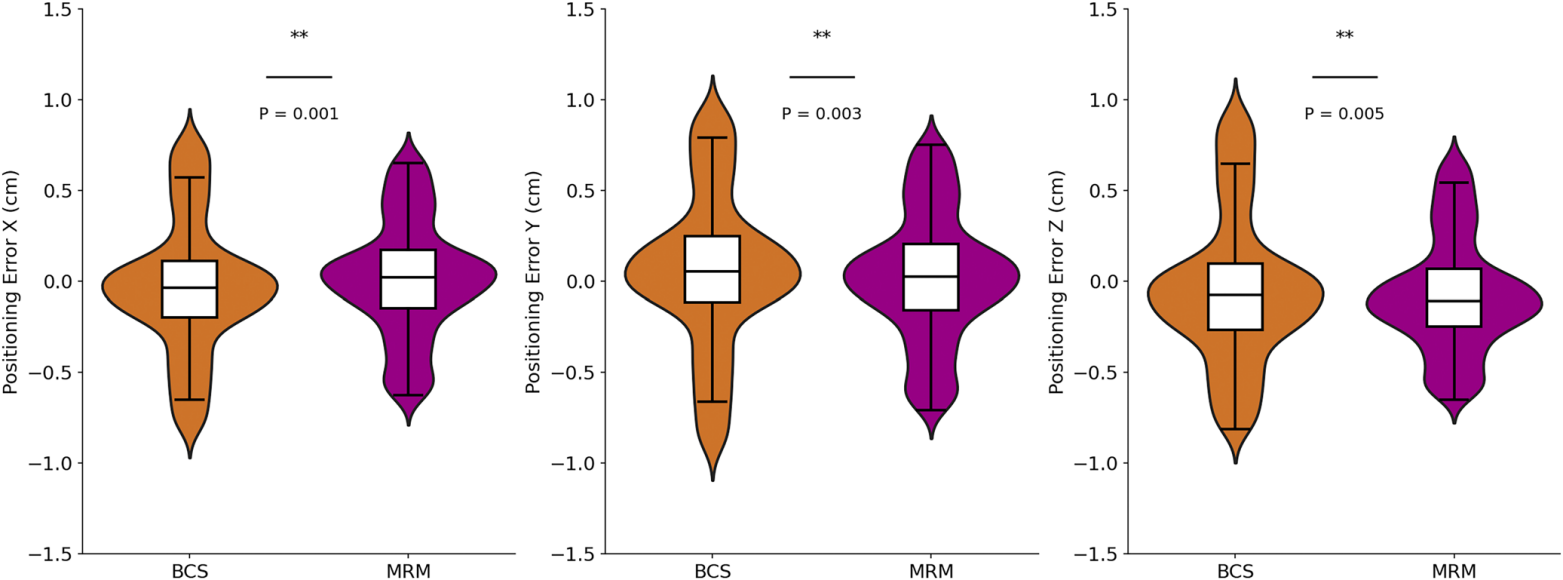
The translational error distribution of patients with different surgery types.

**FIGURE 4:**
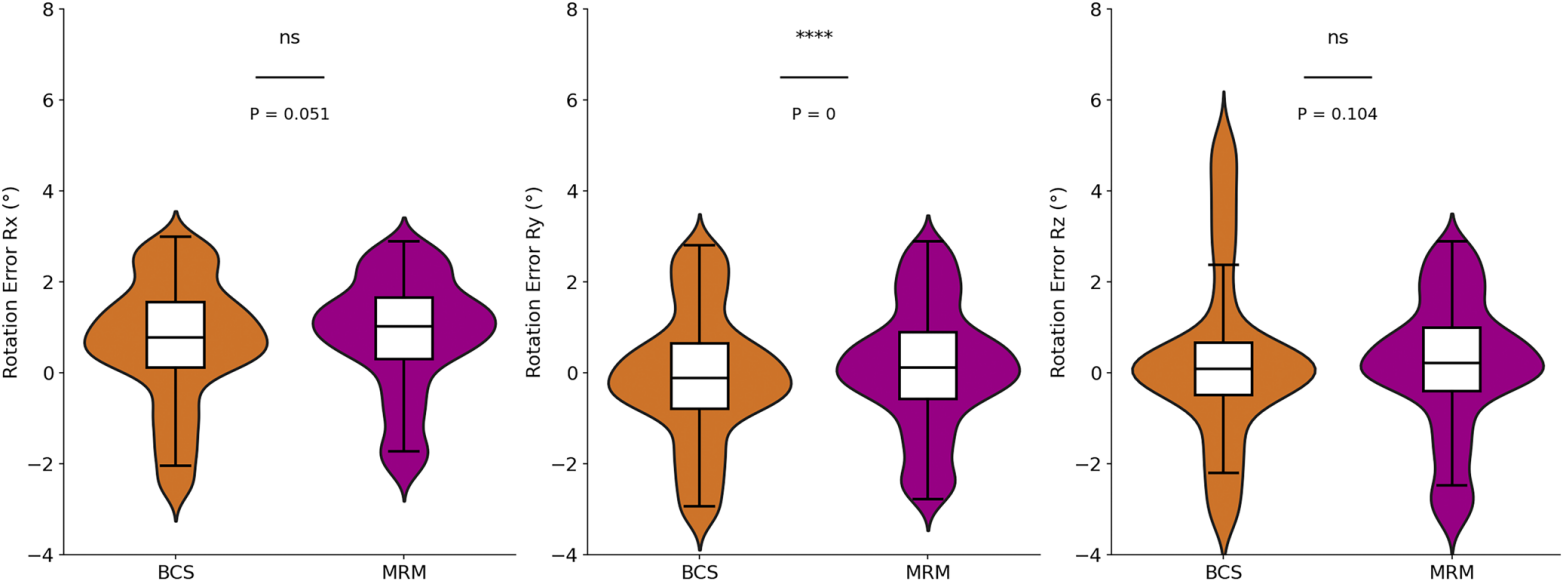
The rotational error distribution of patients with different surgery types.

Figure 3 presents the positioning error data grouped by different surgical types (BCS or MRM). The analysis reveals notable differences between the two groups. In the translational error directions, the BCS group exhibits a broader range of errors, with maximum values of 0.8, 0.96, and 0.95 cm, respectively, compared to the MRM group, where the maximum values are 0.69, 0.77, and 0.67 cm. Similarly, the minimum values in the BCS group (*X*: −0.82 cm, *Y*: −0.92 cm, *Z*: −0.83 cm) are lower than those in the MRM group (*X*: −0.66 cm, *Y*: −0.72 cm, *Z*: −0.65 cm). The distributions reflect greater variability in the BCS group. In terms of rotational errors, the BCS group also demonstrates a significantly wider range, especially in the *R*_*x*_ direction, as shown in Figure 4.

By combining the positioning error data of the BCS and MRM groups with the *t*‐test results (Table 3), a more precise analysis of the differences between the two groups can be made. In terms of translational errors, the *t*‐test shows a significant difference in the *X* direction (*t* = −3.31, *p* < 0.05), indicating that the positioning errors in the *X* direction for the BCS group are significantly lower than those of the MRM group. This is consistent with the differences in the distribution of maximum and minimum values, as the BCS group has a larger error range in the *X* direction but lower concentration.

**TABLE 3:** Statistical analysis results of different breast cancer surgery types.

DIR	BCS		MRM		Statistics	
	Mean	STD	Mean	STD	*t*	*p*
*X* (cm)	−0.034	0.249	0.005	0.233	−3.31	< 0.05
*Y* (cm)	0.055	0.294	0.015	0.251	2.98	< 0.05
*Z* (cm)	−0.067	0.282	−0.102	0.214	2.82	< 0.05
*R*_*x*_ (°)	0.862	1.065	0.960	0.952	−1.95	0.05
*R*_*y*_ (°)	−0.075	1.164	0.143	1.025	−4.02	< 0.05
*R*_*z*_ (°)	0.137	1.078	0.222	1.031	−1.62	0.10

In terms of rotational errors, the *t*‐test shows that the difference in the *R*_*x*_ direction is close to the level of significance (*t* = −1.95, *p* = 0.051), indicating that the error differences in the *R*_*x*_ direction between the two groups are small but exhibit a certain trend. The *t*‐test result for the *R*_*y*_ direction (*t* = −4.02, *p* < 0.05) shows that the MRM group has significantly lower errors than the BCS group, which is consistent with the larger minimum values and broader distribution in the *R*_*y*_ direction. The *t*‐test result for the *R*_*z*_ direction (*t* = −1.62, *p* > 0.05) shows that there is no significant difference in the errors between the two groups in this direction. However, when considering the actual data, the maximum error for the BCS group in the *R*_*z*_ direction (5.4°) is significantly higher than the MRM group (2.9°), indicating that extreme values in the BCS group may have a larger impact on the error range.

### 3.3 Positioning Errors of Patients With Different BMIs

**FIGURE 5:**
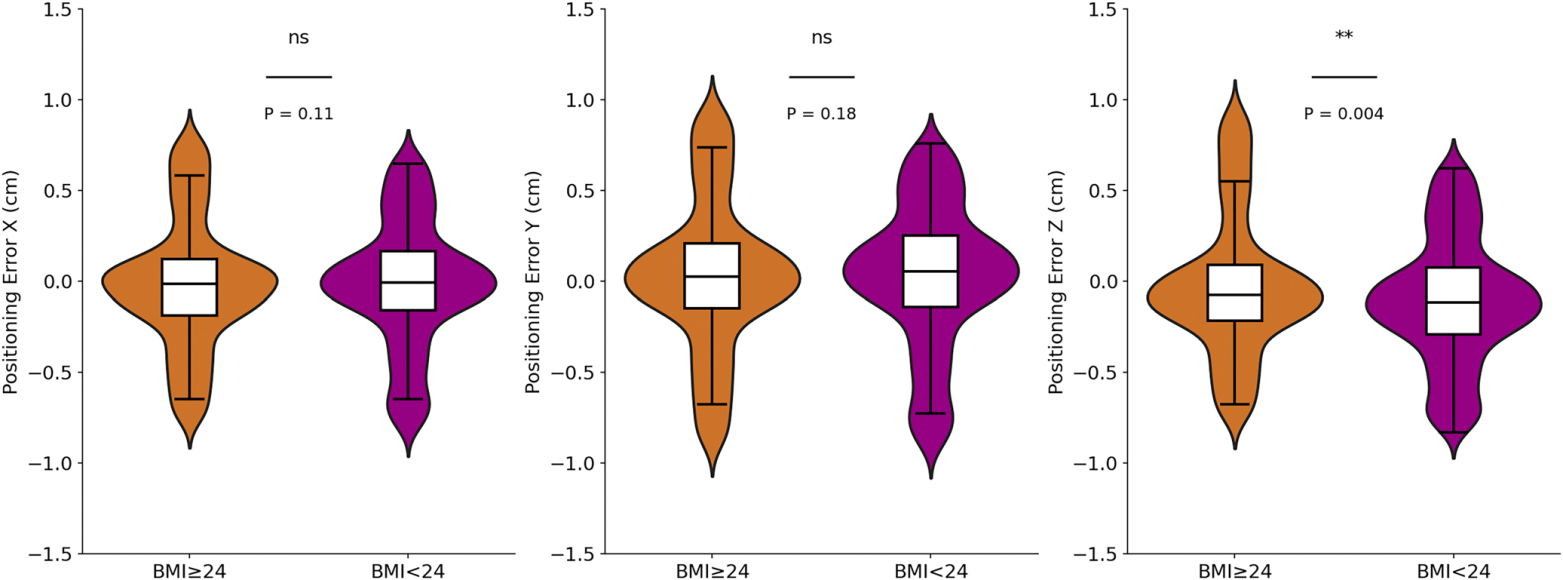
The translational error distribution of patients with different BMIs.

When comparing the positioning error data of breast cancer patients grouped by different BMI categories in Figure 5, significant differences in error were observed across various directions. For the *X* direction, the maximum error for patients with BMI > 24 kg/m^2^ was 0.8 cm, slightly higher than the 0.69 cm observed in patients with BMI < 24 kg/m^2^. In the *Y* direction, the maximum error for BMI > 24 kg/m^2^ patients was 0.96 cm, greater than the 0.76 cm for the BMI < 24 kg/m^2^ group, highlighting a larger positioning error in the superior to inferior direction for patients with higher BMI. Similarly, in the *Z* direction, the maximum error for BMI > 24 kg/m^2^ patients was 0.95 cm compared to 0.64 cm for BMI < 24 kg/m^2^ patients, further demonstrating a greater error trend in this group.

**FIGURE 6:**
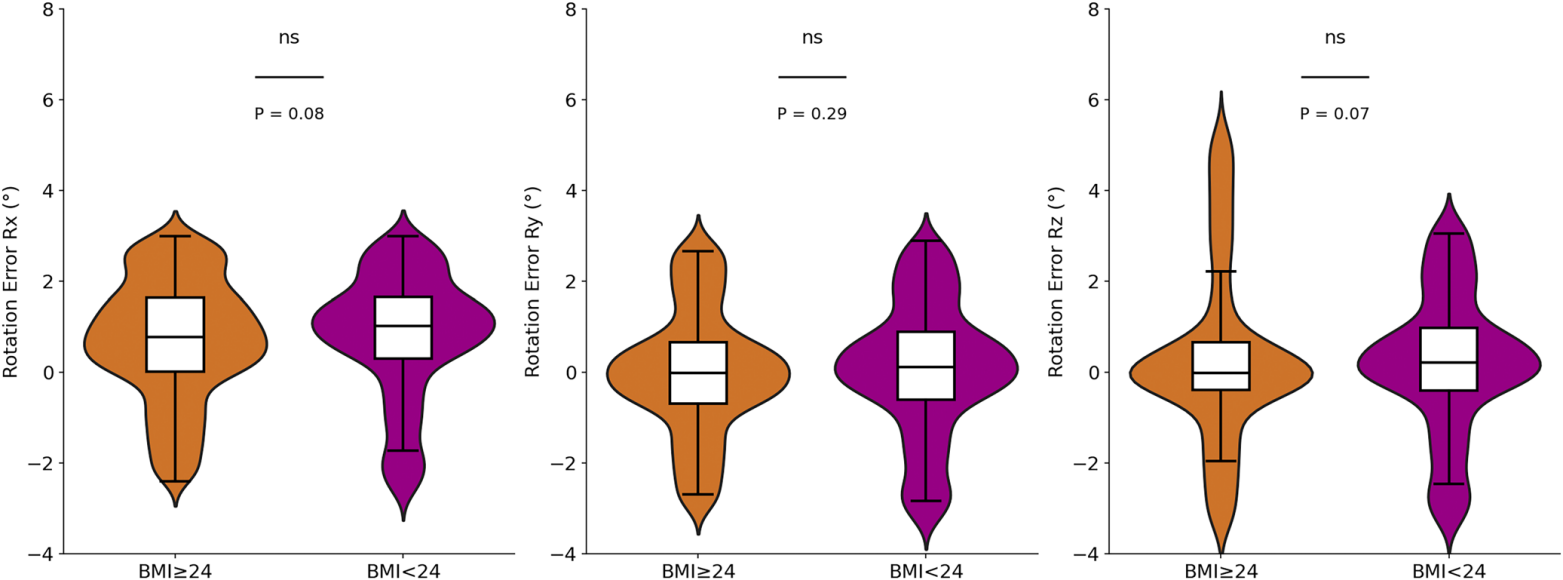
The rotational error distribution of patients with different BMIs.

Regarding rotational errors, patients with BMI > 24 kg/m^2^ exhibited larger errors in the *R*_*x*_, *R*_*y*_, and *R*_*z*_ directions, with the maximum error in the *R*_*z*_ direction reaching 5.4°, significantly exceeding the 3.3° observed in the BMI < 24 kg/m^2^ group. This suggests that patients with higher BMI have greater deviations in rotational errors, as shown in Figure 6.

According to the *t*‐test results in Table 4, there is a significant difference in positioning errors only in the *Z* direction between breast cancer patients with BMI > 24 kg/m^2^ and those with BMI < 24 kg/m^2^, while differences in the *X* and *Y* directions are not statistically significant.

**TABLE 4:** Statistical results of patients with different BMIs in six DOFs.

	BMI < 24 kg/m^2^		BMI ≥ 24 kg/m^2^		Statistics	
	Mean	STD	Mean	STD	*t*	*p*
*X* (cm)	−0.006	0.242	−0.025	0.243	−1.61	0.11
*Y* (cm)	0.044	0.271	0.026	0.278	−1.35	0.18
*Z* (cm)	−0.117	0.251	−0.049	0.248	5.49	< 0.05
*R*_*x*_ (°)	0.951	0.951	0.865	1.065	−1.76	0.08
*R*_*y*_ (°)	0.058	1.077	0.002	1.131	−1.05	0.29
*R*_*z*_ (°)	0.225	1.048	0.127	1.062	−1.84	0.07

## 4 Discussion and Conclusion

In this study, 213 breast cancer patients were grouped based on three factors: tumor site, surgery type, and BMI. Data obtained from these groups were analyzed to evaluate the impact of these factors on positioning errors.

For tumor location, significant setup error differences were observed in all directions except the *Z* direction (*p* < 0.05), suggesting that different tumor locations may significantly affect the stability of setup effect. The quartile range showed that the distribution range of patients with left breast cancer in the *X* direction was −0.24 to 0.07 cm, and the distribution range of patients with right breast cancer was −0.08 to 0.19 cm, indicating that the distribution of patients with left breast cancer was more concentrated, while the variability of patients with right breast cancer was slightly larger. In addition, both patient groups exhibited relatively small mean setup errors in translational directions, whereas the range of rotational errors was relatively larger, particularly in the *R*_*x*_ direction. The median and interquartile range of *R*_*x*_ in right‐sided breast cancer patients remained consistently higher, indicating a greater need for rotational adjustment during setup. In clinical practice, special attention should be paid to the correction accuracy in the *R*_*x*_ direction. Further analysis showed that the significant differences in *t*‐values for *X*, *R*_*y*_, and *R*_*z*_ also help reveal the presence of systematic deviations during the setup process in right‐sided breast cancer patients. This phenomenon may be related to multiple clinical and technical factors. For left‐sided breast cancer, due to the proximity to the heart, DIBH [10] is commonly applied to optimize dose distribution and improve treatment accuracy, which may partially explain the differences observed between the two sides. On the other hand, the use of arm supports in immobilization devices may introduce asymmetry between the left and right sides due to variations in reproducibility among certain patients. It is recommended to optimize the simulation positioning process and increase the frequency of IGRT for right‐sided breast cancer patients to ensure setup reproducibility.

For patients undergoing different surgical types (BCS and MRM), the *t*‐test results confirmed significant differences in positioning errors between the two groups, particularly in the *Y* and *Z* translational directions and the *R*_*y*_ rotational direction. The *t*‐test for the *Y* direction (*t* = 2.98, *p* < 0.05) shows that the BCS group has significantly higher positioning errors in the *Y* direction, while the results for the *Z* direction (*t* = 2.82, *p* < 0.05) show a similar trend, suggesting that the positioning errors in the *Y* and *Z* directions for the BCS group may be more pronounced due to the influence of residual breast tissue, requiring special attention during positioning correction. The BCS group exhibited a broader error distribution and significant differences in some directions, likely attributable to changes in residual breast tissue morphology post‐surgery and increased positioning complexity. Conversely, the MRM group showed a narrower error range and higher concentration, reflecting greater stability in chest wall positioning due to complete breast tissue removal. Clinically, these differences suggest that the BCS group requires more individualized correction strategies, such as increasing CBCT imaging frequency, using more refined immobilization devices, or adopting patient‐specific positioning systems to enhance treatment precision and reduce positioning error impact. For the MRM group, maintaining adherence to positioning protocols remains essential to ensure treatment safety and accuracy.

Analysis of positioning errors for patients with BMI > 24 kg/m^2^ and BMI ≤ 24 kg/m^2^ provided additional insights. No significant differences were observed in the *X* and *Y* directions, suggesting that while higher BMI patients may exhibit slightly larger errors in these directions, the differences are insufficient to reach statistical significance, likely due to random factors like body shape. In contrast, the *Z* direction showed a significant difference (*p* < 0.05), with higher BMI patients exhibiting greater errors. Median values further support this trend, as errors in the BMI > 24 kg/m^2^ group were generally higher than those in the BMI < 24 kg/m^2^ group, particularly in the *Z* direction and *R*_*z*_ rotation. This result is in agreement with previous studies [11]. These findings indicate that patients with higher BMI may require additional attention and adjustments during positioning to ensure treatment accuracy in radiotherapy. This may be explained by the increased presence of adipose tissue in higher BMI patients, particularly in the abdominal and chest regions, contributing to larger displacement errors due to body size and weight changes, especially in the vertical (*Z*) direction. Clinically, this highlights the need for additional adjustments to ensure precise positioning for high BMI patients.

Rotational error analysis showed a trend toward differences in the *R*_*z*_ direction, but *p* values remained above 0.05, indicating no statistical significance. Rotational errors are influenced by factors such as posture, respiration, and equipment stability, which may contribute similarly to errors in both groups. Further accumulating more data could provide more robust conclusions.

In summary, for breast cancer patients with tumors located in different sites, directions with smaller errors and no significant differences (such as the *Z*‐axis) can be used as a reference standard to further identify process differences in positioning errors across other directions. For patients undergoing breast‐conserving surgery, more individualized correction strategies during positioning are necessary, such as increasing the frequency of CBCT imaging or utilizing surface‐guided radiation therapy (SGRT) to further enhance treatment accuracy [12]. For obese patients, optimization measures should be taken to address vertical positioning errors based on body shape differences, including changes in body size among the treatment fractions. These measures may involve adjusting the posture, optimizing immobilization devices, and enhancing posture monitoring to reduce vertical positioning deviations caused by body shape variations.

## Author Contributions

Bao Wan and Fukui Huan conceived of the study design and analysis. Yandong Ge, Ruiao Zhao, Yongtai Zheng, Meng Liang, Lu Hou and Hongkai Wang performed data measurement and analysis, and drafted the manuscript. Yanxin Zhang, Wei Zhang, Ye Zhang and Hongkai Wang coordinated the study and participated in discussions of the manuscript. All authors read and approved the final manuscript.

## Conflicts of Interest

The authors declare no conflicts of interest.

## Data Availability

The datasets generated and analyzed during the current study are available from the corresponding authors with reasonable request.

## References

[1] National Cancer Center/National Cancer Quality Control Center , “Guideline of Target Delineation and Treatment Planning of Adjuvant Radiotherapy for Breast Cancer,” Chinese Journal of Radiation Oncology 31, no. 10 (2022): 863–878, 10.3760/cma.j.cn113030-20220627-00226.

[2] G.‐E. Laura , C. Javier , P. Silvia , C. Isabel , G. Isabel , and M.‐B. Gema , “Obesity and Breast Cancer: Paradoxical and Controversial Relationship Influenced by Menopausal Status,” Frontiers in Oncology 11 (2021): 705911, 10.3389/fonc.2021.705911. 34485137 PMC8414651

[3] Y. Zhao , Q. Liu , H. Lu , et al., “A Non‐Delay Error Compensation Method for Dual‐Driving Gantry‐Type Machine Tool,” PRO 8, no. 7 (2020): 748, 10.3390/pr8070748.

[4] J. Deng , Y. Huang , X. Wu , Y. Hong , and Y. Zhao , “Comparison of Dosimetric Effects of MLC Positional Errors on VMAT and IMRT Plans for SBRT Radiotherapy in Non‐Small Cell Lung Cancer,” PLoS One 17, no. 12 (2022): e0278422, 10.1371/journal.pone.0278422. 36454884 PMC9714892

[5] K. Wada , C. Nagata , A. Tamakoshi , et al., “Body Mass Index and Breast Cancer Risk in Japan: A Pooled Analysis of Eight Population‐Based Cohort Studies,” Annals of Oncology 25, no. 2 (2014): 519–524, 10.1093/annonc/mdt565. 24412821

[6] L. Li and Y. Li , “Effect of Body Mass Index on Inter‐Fractional Positioning Errors in Cone Beam CT‐Guided Radiotherapy of Cervical Cancer,” Chinese Journal of Medical Physics 40, no. 2 (2023): 144–148, 10.3969/j.issn.1005-202X.2023.02.003.

[7] S.‐J. Wang , Y.‐R. Zhai , W.‐W. Zhang , et al., “Dosimetric Benefit and Clinical Feasibility of Deep Inspiration Breath‐Hold and Volumetric Modulated Arc Therapy‐Based Postmastectomy Radiotherapy for Left‐Sided Breast Cancer,” Scientific Reports 14, no. 1 (2024): 24638, 10.1038/s41598-024-75560-5. 39428424 PMC11491445

[8] S. Dubey , K. Krishnanand , Y. Shukla , et al., “Factors Influencing Surgical Choices in Breast Cancer Treatment in India: A Comparative Study of Breast‐Conserving Surgery vs Mastectomy,” Cureus 16, no. 8 (2024): e66825, 10.7759/cureus.66825. 39280530 PMC11393521

[9] National Health Commission of the People's Republic of China , “Guidelines for the Diagnosis and Treatment of Obesity (2024 Edition),” Chinese Journal of Digestive Surgery 23, no. 10 (2024): 1237–1260.

[10] J. Lai , Z. Luo , H. Hu , et al., “SGRT‐Based DIBH Radiotherapy Practice for Right‐Sided Breast Cancer Combined With RNI: A Retrospective Study on Dosimetry and Setup Accuracy,” Journal of Applied Clinical Medical Physics 24, no. 8 (2023): e13998, 10.1002/acm2.13998. 37087557 PMC10402663

[11] I.‐C. Costin and L. G. Marcu , “Impact of Immobilization System Angle, Body Mass Index and Breast Size on Breast Radiotherapy Accuracy Using EPID‐Only Setup,” Heliyon 11, no. 3 (2025): e42176, 10.1016/j.heliyon.2025.e42176. 39959482 PMC11830291

[12] M. Laaksomaa , A. Aula , S. Sarudis , et al., “Surface‐Guided Radiotherapy Systems in Locoregional Deep Inspiration Breath Hold Radiotherapy for Breast Cancer ‐ a Multicenter Study on the Setup Accuracy,” Reports of Practical Oncology and Radiotherapy: Journal of Greatpoland Cancer Center in Poznan and Polish Society of Radiation Oncology 29, no. 2 (2024): 176–186, 10.5603/rpor.99673. 39143974 PMC11321775

